# Biomechanical Properties of Novel Porous Scaffold Core and Hollow Lateral Hole Pedicle Screws: A Comparative Study in Bama Pigs

**DOI:** 10.1111/os.14091

**Published:** 2024-05-20

**Authors:** Yong Hu, Xijiong Chen, Zhentao Chu, Linwei Luo, Zhiwei Gan, Jianbin Zhong, Zhenshan Yuan, Bingke Zhu, Weixin Dong

**Affiliations:** ^1^ Department of Spine Surgery Ningbo No. 6 Hospital Ningbo China; ^2^ Health Science Center Ningbo University Ningbo China

**Keywords:** Animal Surgery, Biomechanical Test, Pedicle Screw, Porous Scaffold, Screw Loosening

## Abstract

**Objective:**

Screw loosening is a common complication of internal fixation of pedicle screw. Therefore, the development of a pedicle screw with low loosening rate and high biosafety is of great clinical significance. This study aimed to investigate whether the application of a porous scaffold structure can improve the stability of pedicle screws by comparing the biomechanical properties of novel porous scaffold core pedicle screws (PSCPSs) with those of hollow lateral hole pedicle screws (HLHPSs) in a porcine lumbar spine.

**Methods:**

Thirty‐two pedicle screws of both types were implanted bilaterally into the L1–4 vertebrae of four Bama pigs, with our newly designed PSCPSs on the right and HLHPSs on the left. All the Bama pigs were sacrificed 16 weeks postoperatively, and the lumbar spine was freed into individual vertebrae. Biomechanical properties of both the pedicle screws were evaluated using pull‐out tests, as well as cyclic bending and pull‐out tests, while the mechanical properties were assessed using three‐point bending tests. The data generated were statistically analyzed using paired‐sample *t*‐tests and two independent sample *t*‐tests.

**Results:**

We found that the maximal pull‐out forces before and after cyclic bending of the PSCPSs (1161.50 ± 337.98 N and 1075.25 ± 223.33 N) were significantly higher than those of the HLHPSs (948.38 ± 194.32 N and 807.13 ± 242.75 N) (*p* < 0.05, *p* < 0.05). In 800 cycles of the bending tests, neither PSCPS nor HLHPS showed loosening or visible detachment, but their maximal pull‐out forces after cyclic bending tests decreased compared to those in cycles without cyclic bending tests (7.43% and 14.89%, respectively), with no statistical significance (*p* > 0.05 and *p* > 0.05, respectively). Additionally, both screws buckled rather than broke in the three‐point bending tests, with no statistically significant differences between the maximal bending load and modulus of elasticity of the two screws (*p* > 0.05 and *p* > 0.05, respectively).

**Conclusions:**

Compared with the HLHPSs, the PSCPSs have greater pull‐out resistance and better fatigue tolerance with appropriate mechanical properties. Therefore, PSCPSs theoretically have significant potential for clinical applications in reducing the incidence of loosening after pedicle screw implantation.

## Graphical abstract

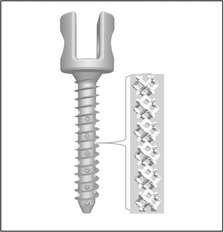

## Introduction

The pedicle screw system has been widely used to treat spinal degeneration, spinal stenosis, fractures, deformities, and tumors,^1^, ^2^, ^3^ and it has achieved excellent treatment results. However, screw loosening is a common complication of internal fixation surgery.^4^ Loosening of pedicle screws can lead to loss of integrity of the screw‐bone interface.^5^ This can seriously affect surgical outcomes, cause pain, and, in severe cases, lead to paralysis or even death.^6^ Additionally, revision surgery due to screw loosening has negative physical, psychological, and economic consequences for patients.

The incidence of screw loosening has been reported to be between 0.6% and 11% in patients with normal bone density and up to 60% in patients with osteoporosis.^7^, ^8^ To reduce the incidence of screw loosening, several studies have been conducted on various aspects, such as modifying the screw design, adding surface coatings, optimizing the screw placement, and filling with bone cement to obtain better mechanical bonding at the screw‐bone interface.^6^, ^9^ However, the practical application of these strategies is limited by a number of complications, such as the risk of leakage, pulmonary embolism, and toxic reactions with the use of bone cement reinforcement.^10^, ^11^ Larger screws increase the risk of pedicle fracture, and longer screws tend to penetrate the vertebral body, leading to vascular rupture and visceral injury.^12^, ^13^ Therefore, developing a pedicle screw that combines long‐term stability and biosafety is difficult.

In recent years, the development of three‐dimensional (3D) printing technology has introduced new clinical possibilities in the field of tissue engineering. Porous scaffolds fabricated using 3D printing technology not only have precise and adjustable pore sizes and porosities,^14^, ^15^ but can also reduce the modulus of elasticity of implants, thereby mitigating the stress‐shielding effect.^16^ Additionally, studies have shown that porous scaffold structures exhibit good osteoconductivity and effectively promote bone growth and osseointegration.^14^, ^17^ Such porous scaffolds have been studied in the field of oral implantation; however, they are mostly placed on the surface of the implant, and very few studies have been conducted on their application at the center of the implant.

We designed a novel porous scaffold core pedicle screw (PSCPS) to replace the hollow part of a hollow lateral hole pedicle screw (HLHPS) with a porous scaffold. The screw was fabricated in one piece using 3D printing rather than being assembled later. We expected the bone tissue to grow into the centrally located porous scaffold through the lateral holes, thereby increasing the contact area between the cancellous bone and screw.

In this study, the PSCPSs and HLHPSs were implanted into the lumbar vertebrae of live Bama pigs. Anti‐pull‐out strength, fatigue resistance, and mechanical properties of the two screw types were compared using biomechanical and mechanical strength tests. The aim of this study are as follows: (i) to investigate the effect of a porous scaffold structure on the mechanical properties of screws; (ii) to verify whether PSCPSs had superior biomechanical properties compared to HLHPSs; and (iii) to analyze the possible reasons for the difference in biomechanical properties between PSCPSs and HLHPSs. We hypothesized that PSCPSs would provide greater resistance to screw loosening in cancellous bone than HLHPSs because of their increased surface contact with the bone.

## Methods

### Study Design

This was a randomized controlled animal trial. All experiments were approved by the Animal Ethics and Welfare Committee of the Zhejiang Chinese Medical University (Approval No: IACUC‐20221205‐02). This study followed the Principles of Laboratory Animal Care (NIH Publication Vol 25, No. 28 revised 1996; http://grants.nih.gov/grants/guide/notice-files/not96-208.html).

### Design of Implanted Screws

**FIGURE 1:**
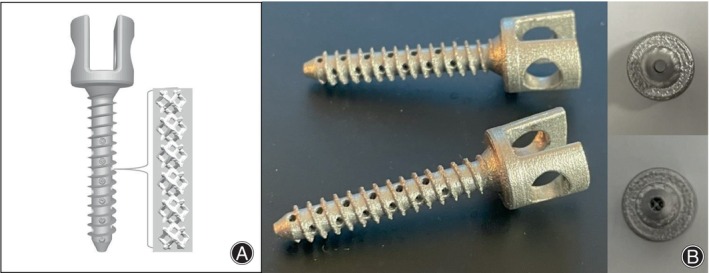
Novel porous scaffold core pedicle screw (PSCPS) and hollow lateral hole pedicle screw (HLHPS). (A) CAD design rendering of PSCPS; (B) lateral (left) and top (bottom right) views of PSCPSs and top (top right) view of HLHPSs, which share the same lateral view as PSCPSs.

In order to increase the contact area of the screw with cancellous bone by means of a porous scaffold and thus improve the stability after implantation, we designed PSCPSs (Patent No. ZL 2022 21,811,701.X). Self‐designed PSCPSs were made of a titanium alloy measuring 4.8 × 25 mm and consisted of an HLHPS shell and porous scaffold core (Figure 1). The lateral holes were distributed at the front of the nail body, with a diameter of 1.1 mm, and one lateral hole was set at every other pitch, totaling 29 lateral holes. The porous scaffold core had a diameter of 1.6 mm, length of 25 mm, pore shape of rhombic dodecahedron, pore diameter of 600 μm, and porosity of 70%. The control group used HLHPSs with the same specifications and lateral pore distributions, which were hollow inside (Figure 1). Both screws were produced using 3D printing by the Shandong Weigao Orthopedic Medical Devices Company.

### Animal Surgery

Four healthy adult male Bama pigs (age, 2–3 years; weight, 52 ± 1 kg) provided by the Taizhou Taihe Biotechnology Company Limited (Jiangsu, China) were used in the experiments. Deformities, fractures, and other lesions were excluded using X‐ray examination. Surgery was performed by a spine surgeon and three assistants. Thirty‐two pedicle screws comprising two types were autoclaved and implanted into the bilateral pedicles of the L1–4 vertebrae of the four Bama pigs. Eight pedicle screws, four PSCPSs on the right side, and four HLHPSs on the left side were used in each Bama pig.

**FIGURE 2:**
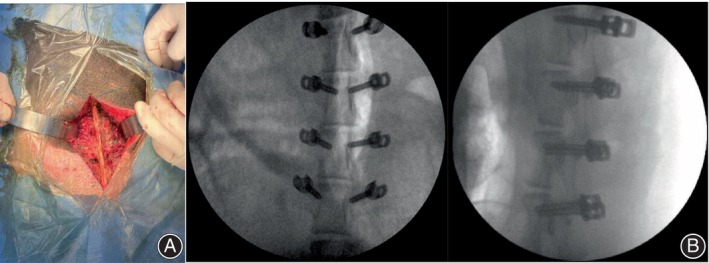
Implantation of pedicle screws. (A) Implantation of novel porous scaffold core pedicle screws (PSCPSs) on the right side of the lumbar spine and hollow lateral hole pedicle screws (HLHPSs) on the left side of the lumbar spine of Bama pigs; (B) frontal and lateral radiographs of the lumbar spine after implantation of the PSCPSs and HLHPSs.

Preoperatively, the Bama pigs were fasted for 12 h and then deprived of water for 4 h. Atropine (2 mg/kg) and propofol (4 mg/kg) were used for induction of anesthesia, and 3% isoflurane was used to maintain general anesthesia via a ventilator. The pigs were placed in the prone position and sterilized after skin preparation. A longitudinal midline incision was made to separate the muscles and expose the lumbar spine. Bilateral pedicle screw fixation was performed after positioning with a localization needle (Figure 2A). The positions of the pedicle screws were confirmed using a C‐arm machine (PLX7000B; Nanjing PUAI Radiographic Imaging Equipment Company, Ltd.) (Figure 2B). After a successful screw placement, the incision was irrigated with saline, vancomycin powder was sprinkled on it, and the incision was closed layer‐by‐layer. Cefaclor was administered orally for 7 days postoperatively to prevent infection. Sixteen weeks after the surgery, the four Bama pigs were anesthetized using an overdose of 3% pentobarbital (3 mg/kg) and subsequently euthanized by bloodletting. After euthanizing the pigs, the lumbar vertebrae were removed intact, and the surrounding soft tissues were removed, dissected from the intervertebral discs, and freed into individual vertebrae. Carcasses of the Bama pigs were handed over to the Laboratory Animal Center for safe disposal. Eight vertebrae were randomly selected from all the specimens for the pull‐out tests, and the remaining eight vertebrae were used for the cyclic bending and pull‐out tests of the screws. Another eight each of PSCPSs and HLHPSs were subjected to the three‐point bending tests.

### Pull‐out Tests

**FIGURE 3:**
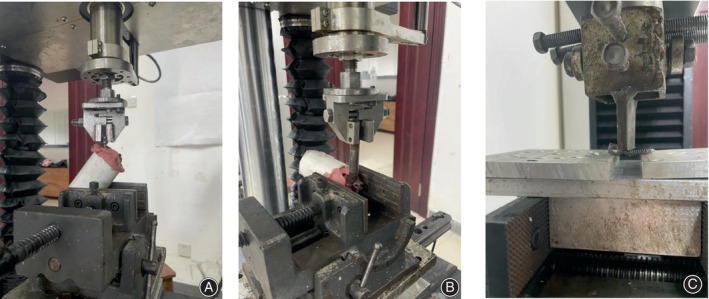
Schematic diagram of biomechanical tests. (A) Pull‐out test. The pull‐out force was similarly aligned with the longitudinal axis of the screw, and the contralateral pedicle screw had been pulled out; (B) cyclic bending test. Loading was applied to the end of the screw through the indenter in a vertical direction to the screw axis; (C) three‐point bending test. The screw‐bone junction was the fulcrum of the test screw.

The samples were removed from the refrigerator 12 h before testing and thawed naturally at room temperature. The lumbar vertebral samples were embedded in a denture base resin. Denture powder had to be used with great care to avoid penetration into the exposed implant and bone interface, as this could enhance the biomechanical strength and affect data reliability. The vertebral specimens were secured to the fixture of an experimental machine (BOSE ElectroForce 3510; MA, USA), and a linear axial extraction force was applied along the longitudinal axis of the pedicle screws (Figure 3A). Each screw was extracted from the pedicle at a constant loading rate of 3 mm/min until fixation failure. The force–displacement curve was continuously captured, and the peak force before failure was recorded as the maximal pull‐out force (Fmax).

### Cyclic Bending and Pull‐out Tests

Cyclic bending tests were performed on the PSCPSs and HLHPSs using the same testing machine used for the pull‐out tests. Each specimen was fixed on the test rig such that the longitudinal axis of the pedicle screw was parallel to the test rig surface. A load was applied to the tail of the pedicle screw in the form of a sine wave from the indenter perpendicular to the screw (Figure 3B). The loading frequency was set at 1.5 Hz, and starting from a base load of 50 N, the load was gradually increased to 300 N and then decreased to 50 N (50 N → 300 N → 50 N), a process that was carried out 800 times. After 800 loading cycles, the samples were reoriented, pull‐out tests were performed on the pedicle screws at a loading speed of 3 mm/min, and Fmax was recorded.

### Three‐Point Bending Tests

After determining the implantation depth of the post‐extraction screws, the site of this depth was marked on all new screws to be tested. The pedicle screws were fixed in an electronic universal testing machine (ATES6010, Guangzhou Aojin Industrial Automation System Company Limited) for compression fracture testing (Figure 3C), and the marked location was considered the pivot point of the test screws. The load was applied at a rate of 3 mm/min, and was stopped when the screws bent or fractured. The maximum bending load and modulus of elasticity, which were automatically captured by the software, were recorded.

### Statistical Analysis

Data were analyzed using SPSS software (version 26.0; IBM, Armonk, NY, USA). Variables were expressed as mean ± standard deviation. Pull‐out data for the two screws were compared using a paired‐sample *t*‐test. Comparisons of the maximal bending load, modulus of elasticity, and pull‐out data before and after the cyclic bending resistance were performed using two independent sample *t*‐tests. Statistical significance was set at *p* < 0.05.

## Results

### Animal Surgery

All the screws were successfully implanted into the bilateral pedicles of the lumbar spine of the Bama pigs, and no failed screws required debugging. The four pigs recovered well after the surgery without complications, such as paralysis or incision infection.

### Pull‐out Tests

**FIGURE 4:**
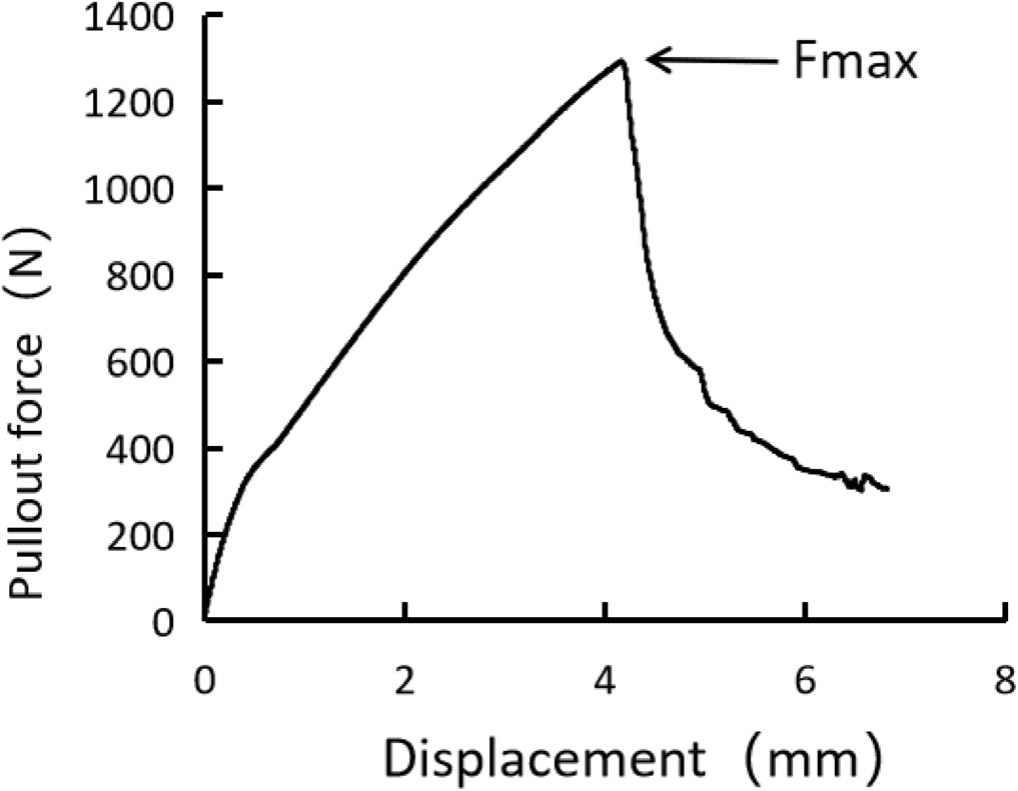
Force‐displacement curve after the axial pull‐out test of the novel porous scaffold core pedicle screw (PSCPS). The maximal pull‐out force (Fmax) was defined as the inflection point at which the load hits its peak and drops sharply with increasing displacement.

**FIGURE 5:**
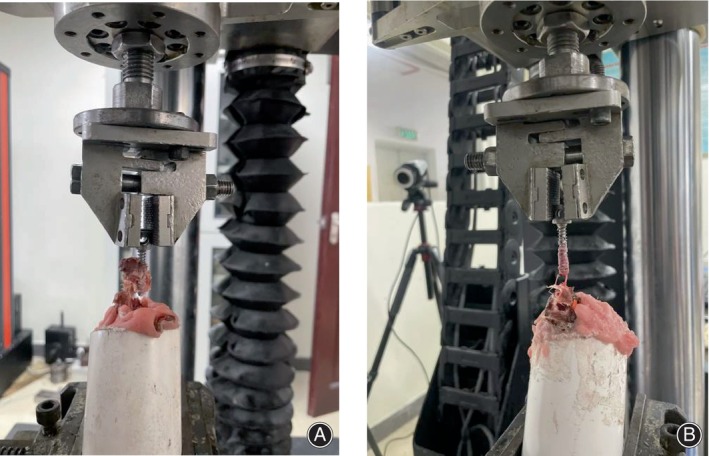
Situation when the pedicle screws were completely extracted. (A) The porous scaffold core pedicle screw (PSCPS) was extracted with a large amount of cancellous bone and fractured pedicle; (B) The hollow lateral hole pedicle screw (HLHPS) was extracted with a small number of bone adhesions (B). Novel porous scaffold core pedicle screws (PSCPSs) had excellent pullout resistance and fatigue tolerance with appropriate mechanical properties. Therefore, PSCPSs theoretically had a significant potential for clinical application in reducing the incidence of loosening after pedicle screw implantation.

The maximum pull‐out force can be measured from the force–displacement curve (Figure 4). During extraction of the PSCPS, a large amount of cancellous bone was attached to the screw surface through the lateral hole, and the cortical bone at the pedicle was fractured and extracted together (Figure 5A). In contrast, only a small quantity of cancellous bone was attached to the surface of the HLHPS (Figure 5B). The Fmax values of the PSCPSs and HLHPSs of the eight vertebrae are listed in Table 1. There was a significant difference between the Fmax of the PSCPSs and HLHPSs at 4 months postoperatively (*p* = 0.04), and the Fmax of the PSCPSs was 22.47% greater than that of the HLHPSs.

**TABLE 1:** Fmax results for PSCPSs and HLHPSs without and after cyclic bending (*N*).

Test	Specimen (*n*)	No.								Means ± SD	*t*	*p*
		1	2	3	4	5	6	7	8			
Pull‐out test	8											
PSCPS		773.00	1047.00	1541.00	1306.00	1116.00	1580.00	636.00	1293.00	1161.50 ± 337.98	2.521	0.04
HLHPS		903.00	638.00	1220.00	1136.00	864.00	1030.00	760.00	1036.00	948.38 ± 194.32		
Cyclic bending and pull‐out test	8											
PSCPS		1384.00	1180.00	688.00	1039.00	967.00	892.00	1246.00	1206.00	1075.25 ± 223.33	2.633	0.034
HLHPS		1069.00	1139.00	958.00	603.00	406.00	796.00	736.00	750.00	807.13 ± 242.75		

Abbreviations: Fmax, maximal pull‐out force; HLHPS, hollow lateral hole pedicle screw; PSCPS, porous scaffold core pedicle screw; SD, standard deviation.

### Cyclic Bending and Pull‐out Tests

After 800 bending test cycles, no loosening or detachment was visible to the naked eye for either pedicle screw. The Fmax values after the cyclic bending of the PSCPSs and HLHPSs are presented in Table 1. The Fmax of the PSCPSs was 33.22% higher than that of the HLHPSs, and there was a statistically significant difference between the two in terms of the pull‐out force (*p* = 0.034). The Fmax values of the PSCPSs and HLHPSs decreased by 7.43% and 14.89%, respectively, after cyclic bending compared to the values in those without cyclic bending, but none of them were statistically significant (*p* > 0.05 and *p* > 0.05, respectively).

### Three‐Point Bending Tests

Both the PSCPSs and HLHPSs were tested with only nail body buckling and no fracture. The maximal bending load and modulus of elasticity of PSCPSs were 408.00 ± 114.28 N and 0.499 ± 0.089 GPa, which were 3.19% and 9.91% higher than those of HLHPSs of 395.38 ± 54.32 N and 0.454 ± 0.058 Gpa, respectively. There was no statistically significant difference between the maximal bending load and modulus of elasticity of the two screws (*p* > 0.05 and *p* > 0.05, respectively).

## Discussion

In the current study, we investigated the biomechanical properties of PSCPSs in preventing postoperative loosening through pull‐out tests as well as cyclic bending and pull‐out tests. The findings indicated that PSCPSs possessed superior pull‐out resistance and fatigue resistance compared to HLHPSs. Therefore, PSCPSs have significant potential for clinical application in reducing the postoperative loosening rate.

### Design Ideas of the PSCPS and Mechanical Properties

With an aging population, diseases, such as lumbar disc herniation, spinal stenosis, and vertebral fractures, have become increasingly common in clinical practice. Internal fixation with pedicle screws is an important surgical procedure for treating these diseases. However, the modulus of elasticity of traditional titanium screws is higher than that of the surrounding bone tissue, which leads to stress shielding, and the surrounding bone is gradually resorbed at a lower stress level for a long period, ultimately triggering aseptic loosening of the pedicle screws.^7^, ^14^ Studies have shown that a porous structure that mimics bone trabeculae can substantially reduce the elastic modulus of an implant, thereby mitigating the effects of stress shielding.^16^, ^18^ Additionally, the bridges of the porous scaffold allow for the creeping deposition of new bone, and the pores facilitate the exchange of nutrients and oxygen during cellular metabolism, while promoting vascularization and bone growth.^19^ However, the generation of pores inevitably leads to a decrease in mechanical strength, and screw breakage triggered by excessive porosity may have serious consequences. Therefore, it is crucial to achieve a balance between biocompatibility and mechanical strength of porous scaffolds. Taniguchi et al.^20^ prepared three types of porous titanium scaffolds with different pore sizes (300, 600, and 900 μm) using 3D printing and implanted them into rabbit cancellous bone for fixation strength testing and bone growth‐in analysis. The results showed that the scaffolds with a pore size of 600 μm were significantly better than the other groups in terms of bonding strength and depth of bone growth at 2 weeks postoperatively. Cheng et al.^21^ found that cell differentiation was best in scaffolds with a porosity of 70%, which was similar to that of human cancellous bone in osteoblast implantation experiments. After reviewing a large number of studies, Gu et al.^22^ stated that porous scaffolds with pore shapes of diamond structure and rhombic dodecahedron could optimally promote osteogenesis, and that porous titanium scaffolds possessing a pore size of 500–600 μm and porosity of 60–70% had mechanical strength comparable to that of human cortical bone. Referring to the conclusions of the above authors, the pore parameters of the porous scaffold core were set as follows in the present study: pore diameter of 600 μm, porosity of 70%, and pore shape of a rhombic dodecahedron, as the conditions for new bone growth and climbing. Additionally, the design of the cortical side holes of the screws spaced one pitch apart rather than more compactly fulfills the dual needs of mechanical strength and adequate windows of bone ingrowth. The PSCPS formed by the combination of the two achieved an excellent match with the vertebral bone. This was confirmed in our study, where the modulus of elasticity of the PSCPSs was 0.499 GPa, which is close to that of cancellous bone (the typical modulus of elasticity of human cancellous bone is 0.4 GPa),^23^, ^24^ offering great potential for improved stress shielding of the surrounding bone and osteoinduction of the implant itself. The increase in the maximal bending load of the PSCPSs compared with that of the HLHPSs suggests that the inner porous scaffold played a role in the increase in the overall mechanical strength of the screw.

### Analysis of Pull‐out Tests Outcomes

Chang et al.^14^ implanted 3D printed porous and solid commercial implants into the distal femur of New Zealand white rabbits, which were subjected to axial compression tests at 4, 8, and 12 weeks postoperatively. The results showed that the ultimate destructive load of the porous implants increased progressively, and was significantly higher than that of commercial implants at 4, 8, and 12 weeks postoperatively (*p* < 0.05). Micro‐computed tomography and histological studies indicated that gradual increase in destructive load correlated with the degree of bone growth, mineralization, and healing tissue maturation. In our study, the Fmax of PSCPSs in the pull‐out tests at 4 months postoperatively increased by 22.47% compared to that of the control group. This was due to the fact that a large amount of newborn bone tissue grew into the porous scaffold and tightly integrated with the pillar in the interconnecting channels, forming a special structure of “bone in screw, screw in bone,” and this structure generated sufficient retention and interlocking forces to maintain the screw stability. The fact that the PSCPSs resulted in a large amount of cancellous bone and led to pedicle fracture during extraction further suggests that the bonding force at the nail‐bone interface was higher than the bone‐bone bonding force. In contrast, the HLHPSs, which lost their porous scaffold cores, resulted in only a small amount of cancellous bone being brought out, indicating that the bonding strength at the nail‐bone interface was not ideal; thus, the pull‐out force was relatively low. However, unlike Chang et al. and most of the designs, our scaffold did not come into direct contact with the bone tissue at the initial stage, but was incorporated inside the pedicle screw, which can increase the utilized volume of the porous scaffold to accommodate more bone growth and improve the screw's long‐term stability. In addition, it can maintain the integrity of the threads and achieve the ability to ensure early fixation of the screw without compromising the screw's overall strength, thereby preventing screw loosening before bone growth. The disadvantage of this design is that cancellous bone takes longer to grow, which increases the possibility of accidents during bone ingrowth.

### Analysis of Cyclic Bending and Pull‐out Tests Outcomes

Pull‐out tests can visually reflect the stability of a screw in its initial state, and Fmax is mainly determined by the shear stress between the screw body and surrounding bone tissue. However, in addition to forces along its long axis, the screw is subjected to transverse bending moments and rotational stresses in vivo. The tail of the screw acts as the site of the transverse bending moment; when stressed, the base of the pedicle becomes a fulcrum. The screw located in the vertebral body presses against the cancellous bone under the effect of leverage, leading to the creation of a small butterfly‐shaped gap between the screw and bone. This “teeter‐totter effect” plays an important role in the development of screw loosening.^25^ In the cyclic bending and pull‐out tests, after 800 cycles of cyclic bending, the pull‐out forces of both screws did not show a significant decrease, indicating that neither screw loosened. However, the decrease in the pull‐out force of the PSCPSs was smaller than that of the HLHPSs, suggesting that the failure tendency of the former was lower. This may be due to the fact that the cancellous bone grew into and clung to the porous scaffold, acting as multiple “bone chains” to anchor the screw. When the screw body was subjected to a leverage force in one direction, the “bone chains” in the other directions would generate stresses to counteract it, thereby alleviating the pressure on the surrounding cancellous bone, which effectively improved the fatigue resistance of the pedicle screw. Additionally, during screw loosening and extraction, the screw was subjected to axial extraction loads while generating spin‐out torque, owing to a thread inclination, and this effect could accelerate the loosening process. For PSCPS, “bone chains” could effectively counteract the spin‐out torque because they were distributed perpendicularly to the longitudinal axis of the screw body, and only a sufficiently large shear force generated by the screw's cortex could break all of the “bone chains” and ultimately lead to screw loosening.

### Study Basis

A previous study^26^ compared the biomechanical properties of hollow lateral holes and solid screws. The results of this study showed that hollow lateral hole screws had significantly better pull‐out and bending resistances than solid screws. Therefore, we believe that PSCPSs have greater fixation strength and long‐term stability than the solid screws used in clinical applications. Its clinical application can reduce the probability of postoperative screw loosening and prevent revision surgery.

### Study Limitations

The present study has some limitations. First, we had only four Bama pigs, which was a relatively small sample size, and we studied only one time point, which did not allow us to explore the effects of different bone growth stages on screw stability and mechanical properties. Second, although pigs and humans have similar lumbar spine structure and bone mineral density,^27^ their walking patterns are different. Therefore, lumbar spine load‐bearing is not the same, and this difference in stress will affect bone growth, resulting in bias in the experimental results. Third, in clinical practice, most patients have varying degrees of osteoporosis; however, we used vertebrae with normal bone conditions, which makes it difficult to realistically reflect the stability of pedicle screws under osteoporotic conditions. Finally, the PSCPS was designed with uniformly spaced lateral holes to maximize the inward growth of new bone tissue; however, this ignored the variations in cancellous bone content in different regions of the vertebrae. In future studies, the design of the lateral hole structure should be improved according to the proportion of cancellous bone in the pedicle and vertebral body.

## Conclusions

Overall, the porous scaffold core of the PSCPS increased the contact area with the cancellous bone after implantation, which not only preserved the immediate anchoring ability of the pedicle screw but also effectively increased the long‐term holding power after implantation and lowered the risk of loosening.

## Author Contributions

All authors had full access to the data in the study and take responsibility for the integrity of the data and the accuracy of the data analysis. YH designed the study and provided a critical review of the manuscript. YH and XJC collected and analyzed the data and wrote the main manuscript. ZTC and LWL provided conceptual advice, statistical analyses, and critically revised the paper. ZSY, JBZ, and BKZ performed the surgeries and collected the data. WXD and ZWG prepared figures and tables and revised the initial manuscript. All authors have read and approved the final submitted manuscript.

## Conflict of Interest Statement

The authors declare that they have no competing interests.

## Funding Information

This study was funded by Medical and Health Research Project of Zhejiang Province (2023KY1147) and Major Special Project of Ningbo Municipal Bureau of Science and Technology (2023Z198).

## References

[1] Nowak B . Experimental study on the loosening of pedicle screws implanted to synthetic bone vertebra models and under non‐pull‐out mechanical loads. J Mech Behav Biomed Mater. 2019;98:200–204. 31260911 10.1016/j.jmbbm.2019.06.013

[2] Varghese V , Krishnan V , Kumar GS . Comparison of pullout strength of pedicle screws following revision using larger diameter screws. Med Eng Phys. 2019;74:180–185. 31543439 10.1016/j.medengphy.2019.09.008

[3] Karakasli A , Acar N , Husemoglu RB . Biomechanical comparison of pullout strengths of six pedicle screws with different thread designs. Jt Dis Relat Surg. 2021;32(1):192–197. 33463436 10.5606/ehc.2021.77004 PMC8073434

[4] Bredow J , Boese CK , Werner CM , et al. Predictive validity of preoperative CT scans and the risk of pedicle screw loosening in spinal surgery. Arch Orthop Trauma Surg. 2016;136(8):1063–1067. 27312862 10.1007/s00402-016-2487-8

[5] Oikonomidis S , Grevenstein D , Yagdiran A , Scheyerer MJ , Eh M , Wegmann K , et al. Probe versus drill: a biomechanical evaluation of two different pedicle preparation techniques for pedicle screw fixation in human cadaveric osteoporotic spine. Clin Biomech (Bristol, Avon). 2020;75:104997. 32335469 10.1016/j.clinbiomech.2020.104997

[6] Rometsch E , Spruit M , Zigler JE , Menon VK , Ouellet JA , Mazel C , et al. Screw‐related complications after instrumentation of the osteoporotic spine: a systematic literature review with meta‐analysis. Global Spine J. 2019;10(1):69–88. 32002352 10.1177/2192568218818164 PMC6963360

[7] Galbusera F , Volkheimer D , Reitmaier S , Berger‐Roscher N , Kienle A , Wilke HJ . Pedicle screw loosening: a clinically relevant complication? Eur Spine J. 2015;24(5):1005–1016. 25616349 10.1007/s00586-015-3768-6

[8] Varghese V , Saravana Kumar G , Krishnan V . Effect of various factors on pull out strength of pedicle screw in normal and osteoporotic cancellous bone models. Med Eng Phys. 2017;40:28–38. 27939099 10.1016/j.medengphy.2016.11.012

[9] Lam TN , Trinh MG , Huang CC , Kung PC , Huang WC , Chang W , et al. Investigation of bone growth in additive‐manufactured pedicle screw implant by using Ti‐6Al‐4V and bioactive glass powder composite. Int J Mol Sci. 2020;21(20):7438. 33050160 10.3390/ijms21207438 PMC7587946

[10] Ulusoy OL , Kahraman S , Karalok I , Kaya E , Enercan M , Sever C , et al. Pulmonary cement embolism following cement‐augmented fenestrated pedicle screw fixation in adult spinal deformity patients with severe osteoporosis (analysis of 2978 fenestrated screws). Eur Spine J. 2018;27(9):2348–2356. 29671110 10.1007/s00586-018-5593-1

[11] Chen LH , Tai CL , Lee DM , Lai PL , Lee YC , Niu CC , et al. Pullout strength of pedicle screws with cement augmentation in severe osteoporosis: a comparative study between cannulated screws with cement injection and solid screws with cement pre‐filling. BMC Musculoskelet Disord. 2011;12:33. 21284883 10.1186/1471-2474-12-33 PMC3224375

[12] Lai DM , Shih YT , Chen YH , Chien A , Wang JL . Effect of pedicle screw diameter on screw fixation efficacy in human osteoporotic thoracic vertebrae. J Biomech. 2018;70:196–203. 29126607 10.1016/j.jbiomech.2017.10.009

[13] Kim YY , Choi WS , Rhyu KW . Assessment of pedicle screw pullout strength based on various screw designs and bone densities‐an ex vivo biomechanical study. Spine J. 2012;12(2):164–168. 22336467 10.1016/j.spinee.2012.01.014

[14] Chang TC , Tsai PI , Chen SY , Kuo MY , Sun JS , Chang JZ . 3D laser‐printed porous Ti(6)Al(4)V dental implants for compromised bone support. J Formos Med Assoc. 2020;119(1 Pt 3):420–429. 31387841 10.1016/j.jfma.2019.07.023

[15] McGilvray KC , Easley J , Seim HB , et al. Bony ingrowth potential of 3D‐printed porous titanium alloy: a direct comparison of interbody cage materials in an in vivo ovine lumbar fusion model. Spine J. 2018;18(7):1250–1260. 29496624 10.1016/j.spinee.2018.02.018 PMC6388616

[16] Cheong VS , Fromme P , Mumith A , Coathup MJ , Blunn GW . Novel adaptive finite element algorithms to predict bone ingrowth in additive manufactured porous implants. J Mech Behav Biomed Mater. 2018;87:230–239. 30086415 10.1016/j.jmbbm.2018.07.019

[17] Ryan G , Pandit A , Apatsidis DP . Fabrication methods of porous metals for use in orthopaedic applications. Biomaterials. 2006;27(13):2651–2670. 16423390 10.1016/j.biomaterials.2005.12.002

[18] Diez‐Escudero A , Andersson B , Carlsson E , Recker B , Link H , Järhult JD , et al. 3D‐printed porous Ti6Al4V alloys with silver coating combine osteocompatibility and antimicrobial properties. Biomater Adv. 2022;133:112629. 35527155 10.1016/j.msec.2021.112629

[19] Pałka K , Pokrowiecki R . Porous titanium implants: a review. Adv Eng Mater. 2018;20(5):1700648.

[20] Taniguchi N , Fujibayashi S , Takemoto M , Sasaki K , Otsuki B , Nakamura T , et al. Effect of pore size on bone ingrowth into porous titanium implants fabricated by additive manufacturing: an in vivo experiment. Mater Sci Eng C Mater Biol Appl. 2016;59:690–701. 26652423 10.1016/j.msec.2015.10.069

[21] Cheng A , Humayun A , Cohen DJ , Boyan BD , Schwartz Z . Additively manufactured 3D porous Ti‐6Al‐4V constructs mimic trabecular bone structure and regulate osteoblast proliferation, differentiation and local factor production in a porosity and surface roughness dependent manner. Biofabrication. 2014;6(4):045007. 25287305 10.1088/1758-5082/6/4/045007 PMC4296567

[22] Gu Y , Sun Y , Shujaat S , Braem A , Politis C , Jacobs R . 3D‐printed porous Ti6Al4V scaffolds for long bone repair in animal models: a systematic review. J Orthopaedic Surg Res. 2022;17(1):68. 10.1186/s13018-022-02960-6 PMC8812248 35109907

[23] Han Q , Wang C , Chen H , Zhao X , Wang J . Porous tantalum and titanium in orthopedics: a review. ACS Biomater Sci Eng. 2019;5(11):5798–5824. 33405672 10.1021/acsbiomaterials.9b00493

[24] Su B , Peng X , Jiang D , Wu J , Qiao B , Li W , et al. In vitro and in vivo evaluations of nano‐hydroxyapatite/polyamide 66/glass fibre (n‐HA/PA66/GF) as a novel bioactive bone screw. PLoS ONE. 2013;8(7):e68342. 23861888 10.1371/journal.pone.0068342 PMC3704538

[25] Jia C , Zhang R , Xing T , Gao H , Li H , Dong F , et al. Biomechanical properties of pedicle screw fixation augmented with allograft bone particles in osteoporotic vertebrae: different sizes and amounts. Spine J. 2019;19(8):1443–1452. 31009768 10.1016/j.spinee.2019.04.013

[26] Hu Y , Chu ZT , Shen SF , Zhong JB , Zhu BK , Wu JD , et al. Biomechanical properties of novel lateral hole pedicle screws and solid pedicle screws: a comparative study in the beagle dogs. Orthop Surg. 2023;15(1):328–336. 36411506 10.1111/os.13596 PMC9837263

[27] Harper RA , Pfeiffer FM , Choma TJ . The minipig as a potential model for pedicle screw fixation: morphometry and mechanics. J Orthopaedic Surg Res. 2019;14(1):246. 10.1186/s13018-019-1292-9 PMC6683400 31382997

